# CircDOCK1 promotes the tumorigenesis and cisplatin resistance of osteogenic sarcoma via the miR-339-3p/IGF1R axis

**DOI:** 10.1186/s12943-021-01453-0

**Published:** 2021-12-07

**Authors:** Shenglong Li, Fei Liu, Ke Zheng, Wei Wang, Enduo Qiu, Yi Pei, Shuang Wang, Jiaming Zhang, Xiaojing Zhang

**Affiliations:** Department of Bone and Soft Tissue Tumor Surgery, Cancer Hospital of China Medical University, Liaoning Cancer Hospital & Institute, Shenyang, 110042 Liaoning Province China

**Keywords:** circDOCK1, miR-339-3p, IGF1R, OS, Cisplatin resistance

## Abstract

**Background:**

Circular RNAs (circRNAs), a class of noncoding RNAs (ncRNAs), may modulate gene expression by binding to miRNAs. Additionally, recent studies show that circRNAs participate in some pathological processes. However, there is a large gap in the knowledge about circDOCK1 expression and its biological functions in osteogenic sarcoma (OS).

**Methods:**

Differentially expressed circRNAs in OS cell lines and tissues were identified by circRNA microarray analysis and quantitative real-time PCR (qRT–PCR). To explore the actions of circDOCK1 in vivo and in vitro, circDOCK1 was knocked down or overexpressed. To assess the binding and regulatory associations among miR-339-3p, circDOCK1 and IGF1R, we performed rescue experiments, RNA immunoprecipitation (RIP), RNA pulldown assays and dual-luciferase assays. Moreover, we performed apoptosis assays to reveal the regulatory effects of the circDOCK1/miR-339-3p/IGF1R axis on cisplatin sensitivity.

**Results:**

CircDOCK1 expression remained stable in the cytoplasm and was higher in OS tissues and cells than in the corresponding controls. Overexpression of circDOCK1 increased oncogenicity in vivo and malignant transformation in vitro. In the U2OS and MG63 cell lines, circDOCK1 modulated tumor progression by regulating IGF1R through sponging of miR-339-3p. Additionally, in the U2OS/DDP and MG63/DDP cell lines, cisplatin sensitivity was regulated by circDOCK1 via the miR-339-3p/IGF1R axis.

**Conclusions:**

CircDOCK1 can promote progression and regulate cisplatin sensitivity in OS via the miR-339-3p/IGF1R axis. Thus, the circDOCK1/miR-339-3p/IGF1R axis may be a key mechanism and therapeutic target in OS.

**Supplementary Information:**

The online version contains supplementary material available at 10.1186/s12943-021-01453-0.

## Background

Osteogenic sarcoma (OS) (also called osteosarcoma) is a primary bone sarcoma that originates from mesenchymal cells and often occurs in children and young adults [1–3]. Approximately 25% of patients present with detectable metastases, most frequently in the lungs [4]. The clinical outcomes of advanced OS remain unsatisfactory, despite the use of combinations of radiotherapy, chemotherapy, and surgery in current treatment regimens [5, 6]. Pre-existing or potential distant metastases result in a high relapse rate in patients. More preclinical modalities (such as targeted therapy) have recently been developed for patients with recurrent OS [7]. However, as a result of the low response rate and serious adverse reactions, there is an urgent need to identify the complex mechanisms underlying the occurrence, chemoresistance and progression of OS.

Circular RNA (circRNA), a newly identified noncoding RNA (ncRNA), is characterized by a distinct single-stranded closed loop structure without a polyadenylated tail or 5′-3′ polarity [8, 9]. Based on accumulating evidence, circRNAs exert their effects mainly through three mechanisms: (1) cis regulation of parental gene expression; (2) microRNA (miRNA) sponging to regulate gene expression (i.e., acting as competitive endogenous RNAs); and (3) formation of complexes with RNA binding proteins (RBPs) [10]. Through these mechanisms, circRNAs are implicated in multiple biological processes, such as proliferation, invasion and apoptosis, and thus participate in tumorigenesis and chemoresistance [11, 12]. Studies have revealed that circRNAs are involved in the pathogenesis of multiple cancers [13], such as breast cancer [14], cholangiocarcinoma [15], and glioblastoma [16]. However, only preliminary studies on the role of circRNAs in OS have been performed [17–19], and a large knowledge gap exists regarding the overall pathophysiological functions of circRNAs in OS.

In this study, through microarray data analysis and quantitative real-time polymerase chain reaction (qRT–PCR), we found that circDOCK1 is highly expressed in OS cell lines and tissues. CircDOCK1 (circBase ID: hsa_circ_0020378) is located on chromosome 10:128594022–128,926,028 and is 2848 nucleotides in length. It enhanced the migration, proliferation and invasion of OS cells. In addition, circDOCK1 modulated cisplatin sensitivity. Thus, circDOCK1 may be a contributor to carcinogenesis and chemotherapeutic resistance by regulating insulin-like growth factor 1 receptor (IGF1R) expression via competitive binding to miR-339-3p.

## Methods

### Clinical specimens

We downloaded microarray-based circRNA expression profiles of 3 primary OS patients (GSE140256) from the GEO database and performed analyses. Seventy pairs of OS tissues and paracancerous tissues were harvested from patients who received complete resection without preoperative chemotherapy at the Cancer Hospital of China Medical University between 2015 and 2019. The definitive diagnosis was made by pathological analysis of the samples. The samples were frozen in liquid nitrogen for 20 min and were then stored at − 80 °C until use. Blood was sampled from 70 normal controls (noncancerous) and 70 OS patients. The blood was naturally agglutinated at room temperature for 60 min, and then centrifuged at 3000 rpm for 5 min to separate the serum. Informed consent forms were obtained from all study subjects. The protocol was approved by the Ethics Committee of the Cancer Hospital of China Medical University.

### Cell culture and transfection

The normal human osteoblastic cell line hFOB 1.19 and human OS cell lines (MG63, SaOS-2, U2OS and HOS) were acquired from the Cell Bank of the Chinese Academy of Sciences (Shanghai, China). DDP-resistant OS cell lines (MG63/DDP, and U2OS/DDP) were established from the parental cell lines MG63 and U2OS by using an intermittent stepwise selection protocol over 6 months. The half maximal inhibitory concentration (IC50) was determined from corresponding dose-response curve. All OS cells were cultured in DMEM (GIBCO, Gaithersburg, MD, USA) supplemented with 10% fetal bovine serum (FBS; GIBCO, Gaithersburg, MD, USA) and incubated at 37 °C in a humidified atmosphere with 5% CO_2_. DMEM/F12 medium (GIBCO, Gaithersburg, MD, USA) was used to culture hFOB 1.19 cells. Moreover, U2OS/DDP and MG63/DDP cells were further treated with 0.5 μg/ml DDP to maintain the resistance. U2OS and MG63 cells were transfected with the sequences listed in Table S1 in Additional file 1 for 24 h using Lipofectamine 2000 (Invitrogen, Carlsbad, CA, USA) for subsequent investigations. For the construction of the circDOCK1 overexpression plasmid, human circDOCK1 cDNA was synthesized and cloned into the pcDNA3.1 vector (Thermo Fisher Scientific, USA). PLKO.1-puro were purchased from BioVector NTCC Inc., Beijing, China. We designed and synthesized an shRNA sequence that targeted circDOCK1 and a negative shRNA control sequence and cloned them into PLKO.1-puro. The siRNAs, miR-339-3p mimics and miR-339-3p inhibitor were purchased from GenePharma (Shanghai, China).

### Ribonuclease R (RNase R) digestion and actinomycin D assay

To verify the circRNA characteristics, 3 μg of RNA was incubated with 20 U/μL RNase R (Epicentre Biotechnologies) for 15 min at 37 °C. Actinomycin D was utilized for the treatment of U2OS and MG63 cells 0, 4, 8, 12 and 24 h before RNA extraction for the detection of DOCK1 and circDOCK1.

### Sanger sequencing

Tsingke (Nanjing, China) performed Sanger sequencing using amplification products of circRNAs in a T vector. Primers (Invitrogen, Shanghai, China) were designed and synthesized to verify the backsplice junction of circDOCK1.

### RNA extraction and qRT–PCR

Total RNA was isolated from cell lines or clinical samples with TRIzol reagent (Invitrogen, Carlsbad, CA, USA). Subsequently, 500 ng of RNA was reverse transcribed into cDNA using a PrimeScript RT Reagent Kit (TaKaRa Bio, Inc., China). We measured the expression of miR-339-3p, circDOCK1 and IGF1R by qRT–PCR and normalized the mRNA and circRNA levels to the GAPDH level and the miRNA level to the U6 level. As shown in Table S2 in Additional file 1, Tsingke (Nanjing, China) synthesized the PCR primer sequences. The fold change in RNA expression was assessed by the 2^−ΔCt^ method.

### Isolation of the cytoplasmic and nuclear fractions

We used NE-PER Nuclear and Cytoplasmic Extraction Reagents (Thermo Fisher Scientific, USA) for the preparation of cytoplasmic and nuclear fractions according to the manufacturer’s instructions. Lysis of OS cells was performed in Lysis Buffer J containing protease inhibitors on ice for 10 min. OS cells were then centrifuged for 3 min at 14,000×g to obtain the precipitate and supernatant as the cytoplasmic and nuclear fractions, respectively, from which RNAs were extracted with Buffer SK and washed with a cleaning solution. Subsequently, the expression of some RNAs was measured by qRT–PCR.

### Fluorescence in situ hybridization (FISH)

FISH was conducted with dedicated probes for miR-339-3p and circDOCK1 and negative control probes according to the product instructions (GenePharma, Shanghai, China). In brief, cells were subjected sequentially to 15 min of fixation with 4% paraformaldehyde (PFA) at indoor temperature, two steps of washing with PBS, and overnight mixing in 70, 95 and 100% ethanol at 4 °C. Next, cells were subjected to overnight hybridization at 37 °C in a humidified chamber in the dark, three steps of washing in saline-sodium citrate buffer for 5 min each, 1 h of incubation in PBS blocking buffer (3% normal goat serum and 1% BSA), and overnight incubation with an anti-biotin antibody at 4 °C. Finally, cell images were acquired under an Olympus BX53 fluorescence microscope (Olympus America, Inc., Center Valley, PA, USA).

### Protein extraction and western blot analysis

RIPA buffer was used for protein extraction. The supernatant of the cell lysate was subjected to SDS–PAGE on 10% acrylamide gels before transfer onto a polyvinylidene difluoride membrane (Millipore). Western blot analysis was conducted with antibodies against IGF1R (1:1000, ab39398, Abcam, Shanghai, China), DOCK1 (1:1000, ab97325, Abcam), AGO2 (1:1000, ab186733, Abcam), GAPDH (1:1000, ab9485, Abcam) and the corresponding HRP-conjugated secondary antibodies (1:1000, Beyotime, Nantong, China), and the emitted light is detected on X-ray films.

### Immunohistochemistry (IHC)

After fixation with 4% PFA, tumor tissues were embedded in paraffin. Sections (5 μm thick) were blocked with 10% goat serum and incubated overnight with an anti-ki67 (1:200, ab16667, Abcam) or anti-IGF1R (1:500, ab39398, Abcam) antibody at 4 °C. Then, images were acquired for further analyses.

### Cell proliferation assay

The proliferation of OS cell lines was measured using a Cell Counting Kit-8 (CCK-8, Dojindo, Osaka, Japan). MG63 and U2OS cells were seeded in 96-well plates, and 10 μL of CCK-8 reagent was added to each well. After another 1 h of incubation at 37 °C, the absorbance was measured at 450 nm using a microplate reader (Bio–Rad). Cell proliferation was evaluated at 0, 24, 48, 72 and 96 h. Each experiment was performed in triplicate.

### 5-Ethynyl-2′-deoxyuridine (EdU) incorporation assay

A Cell-Light EdU DNA Cell Proliferation Kit (RiboBio, Guangzhou, China) was used for the EdU incorporation assay. MG63 and U2OS cells were incubated with 50 mM EdU for 2 h, fixed with 4% PFA, and stained with Apollo Dye Solution. Nuclei were identified by staining with 4′,6-diamidino-2-phenylindole (DAPI). After that, the proliferating cells were imaged and counted under an Olympus FSX100 microscope (Olympus, Tokyo, Japan).

### Colony formation assay

After OS cell lines (MG63 and U2OS) were resuspended at 1 × 10^3^ cells/mL and seeded in 6-well plates, the plates were incubated at 37 °C for 14 days, and the colonies were stained with 20% methanol and 0.1% crystal violet prior to counting.

### Migration and invasion assays

Migration and invasion assays were conducted with Transwell chambers. For invasion assays, 100 μL of Matrigel (BD Bioscience, San Jose, CA, USA) was used to precoat the chamber membranes for 30 min, and the medium was then added into the chambers. OS cells (1 × 10^6^ cells/mL) were resuspended in DMEM after transfection. Subsequently, 100 μL of the cell suspension in serum-free medium was added to the upper chambers, and 600 μL of complete medium was added to the lower chambers. Cells were subjected sequentially to incubation for 24 h in 5% CO_2_ at 37 °C, fixation with 4% PFA and staining with 0.1% crystal violet solution. In five random areas, images were acquired to visualize cells that passed through the filter, and these cells were counted under an inverted fluorescence microscope (Leica Microsystems GmbH, Wetzlar, Germany).

### Animal studies

Female nude mice (6 weeks) obtained from the Laboratory Animal Resources, Chinese Academy of Sciences (Beijing, China) were fed in laminar flow cabinets at room temperature under aseptic conditions on a 12 h light/dark cycle. Food and water were provided ad libitum. A total of 5 × 10^6^ OS cells were subcutaneously implanted into the dorsal surface of mice (6 mice per group). The tumor volume was measured every 7 days and calculated as follows: volume = length × (width/2).^2^ Mice were euthanized 28 days later for tumor resection and collection. For the chemosensitivity assay (6 mice per group), 1 week after cell implantation, 5 mg/kg cisplatin in PBS was administered by intraperitoneal injection t.i.w. The transplanted tumors were collected 4 weeks later. The tail vein injection model was used (6 mice per group) [20]. Ten minutes after 4.0 mg of luciferin (Gold Biotech) in 50 μL of saline was intraperitoneally injected, tumor metastasis was detected with an IVIS@ Lumina II system (Caliper Life Sciences, Hopkinton, MA). The Ethics Committee of the China Medical University approved all experiments.

### Luciferase reporter assay

The circDOCK1 or IGF1R 3′ UTR sequences containing wild-type or mutant miR-339-3p binding sites were synthesized and respectively inserted into pmirGLO luciferase reporters (7350 bp, Promega, Madison, WI, USA) between Sacl and Sall restriction sites, after which cotransfected with miR-339-3p mimics or control mimics into OS cells using Lipofectamine 2000. After the cells were incubated for 48 h, luciferase activity was measured following the instructions (Promega). All experiments were repeated at least three times.

### RNA immunoprecipitation (RIP)

The RIP assay was carried out using a Magna RIP RNA Binding Protein Immunoprecipitation Kit (Millipore) following its instructions; an anti-AGO2 antibody (1:30, ab186733, Abcam) and IgG (1:30, ab109489, Abcam) were used.

### RNA pulldown assay

GenePharma (Shanghai, China) designed and synthesized the biotinylated circDOCK1 probes. These probes were incubated with C-1 magnetic beads (Life Technologies, Waltham, MA, USA) for 2 h at 25 °C to coat the beads. After cell collection and lysis, lysates were incubated overnight with circDOCK1 or oligo probes at 4 °C. A RNeasy Mini Kit was used to pull down RNA-bead complexes. qRT–PCR was performed to assess the abundances of circDOCK1 and miR-339-3p.

### Apoptosis assay

A flow cytometer (FACSCalibur, BD, USA) was used for the apoptosis assay in accordance with the product manual. After treatment for 24 h, washing, resuspension and staining with PI and Annexin V-FITC, the apoptosis rate of cells treated under different conditions was analyzed with the abovementioned flow cytometer. The flow cytometric data were then analyzed with FlowJo V10 software (Tree Star, San Francisco, CA, USA). Each experiment was performed more than three times.

### Cell viability analysis

Transfected and untransfected cells were seeded in 96-well plates (5 × 10^3^ cells/well) for 24 h prior to 48 h of treatment with 0, 0.1, 0.5, 1, 2.5, 5, and 10 μg/mL doxorubicin hydrochloride (DOX) and 0, 1, 2.5, 5, 7.5, 15, and 25 μg/mL cisplatin (DDP). To evaluate the cytotoxicity of cisplatin, cell viability was measured with an MTT Assay Kit (Sigma, St. Louis, MO, USA).

### Statistical analysis

The data are presented as the mean ± standard deviation (SD) values. All statistical analyses were conducted using SPSS 22.0 software (Chicago, Illinois, USA). The significance of between-group differences was assessed by the Mann–Whitney U test or two-tailed Student’s t-test. For normally distributed data, ANOVA with Tamhane’s T2 test (heterogeneous variance) or the S-N-K test (homogeneous variance) were conducted to evaluate differences among groups; for non-normally distributed data, the Kruskal–Wallis test with Dunn’s post hoc test was used. Fisher’s exact test or the chi-square test was conducted to determine whether the associations of circDOCK1 expression with clinicopathological characteristics were significant. Survival curves were estimated by the Kaplan–Meier method, and survival data were compared with the log-rank test. The correlation between circDOCK1 and IGF1R was assessed by Pearson correlation analysis. The threshold for statistical significance was set to *P* < 0.05.

## Results

### Expression and characterization of circDOCK1 in OS cells and tissues

**Fig. 1:**
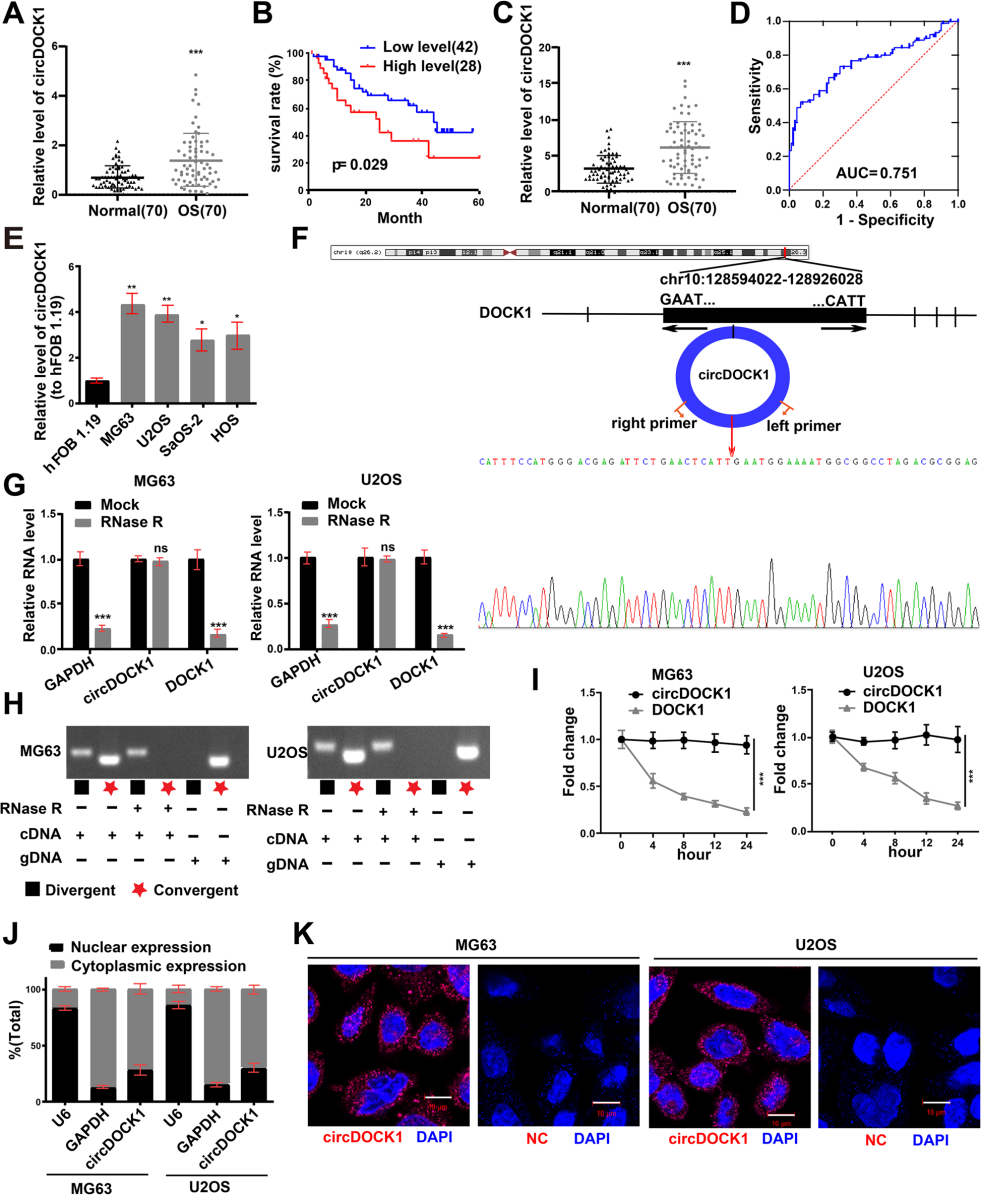
The expression and characterization of circDOCK1 in OS cells and tissues. **A** circDOCK1 expression in OS tissues was measured by qRT–PCR. **B** The association of circDOCK1 expression with overall survival in 70 OS patients was evaluated by Kaplan–Meier analysis. **C** Relative serum circDOCK1 levels in OS patients and non-OS carriers (*n* = 70). **D** Diagnostic value of serum circDOCK1 by ROC curve. **E** CircDOCK1 expression in OS cells (normalized to hFOB 1.19 cells). **F** Sanger sequencing of circDOCK1, with the arrows indicating splice sites. **G** After treatment with RNase R, the abundance of circDOCK1 and linear DOCK1 in U2OS and MG63 cell lines was determined by qRT–PCR (normalized to Mock). **H** qRT-PCR products of linear and circular products amplified with convergent and divergent primers with and without RNase R treatment. **I** qRT–PCR analysis of circDOCK1 and DOCK1 expression in OS cells after actinomycin D treatment. **J** qRT–PCR analysis of isolated cytoplasmic and nuclear fractions. **K** FISH assays using circDOCK1 and negative control probes in the MG63 and U2OS cell lines (scale bar = 10 μm). The data are presented as the mean ± SD of three independent experiments. *P < 0.05, **P < 0.01, ***P < 0.001, ns: nonsignificant

The microarray-based circRNA expression profile GSE140256, containing data for three primary OS patients, was employed to detect the differential expression of circRNAs between noncancerous tissues and OS tissues (Fig. S1A and B in Additional file 2). We analyzed the raw data of GSE140256 with the GEO2R online analysis tool (https://www.ncbi.nlm.nih.gov/geo/geo2r/?acc=GSE140256). Through qRT–PCR, we found that only circDOCK1 showed markedly increased expression in MG63 and U2OS cells compared to hFOB 1.19 cells (Fig. S1C in Additional file 2). CircDOCK1 was chosen for further experiments based on the differential expression of genes in the microarray and OS cell lines. OS tissues showed a significant increase in circDOCK1 expression relative to that in the contiguous normal tissues (Fig. 1A). CircDOCK1 expression in OS tissues was used as the basis for clinicopathological analysis (Table S3 in Additional file 1). Overall survival was unfavorable in OS patients with higher circDOCK1 levels, as indicated by Kaplan–Meier analysis (*P* = 0.029) (Fig. 1B). Then, qRT–PCR showed notably higher circDOCK1 levels in OS serum than in normal serum (Fig. 1C). Furthermore, receiver operating characteristic (ROC) analysis was performed to determine the diagnostic value of circDOCK1 in OS serum, and an area under the ROC curve (AUC) of 0.751 (*P* < 0.001) was obtained (Fig. 1D). Additionally, OS cell lines had higher circDOCK1 levels than hFOB 1.19 cells. We selected MG63 and U2OS cells for further analyses (Fig. 1E).

We evaluated the sequences of the amplification products of circDOCK1 obtained by qRT–PCR with divergent primers by Sanger sequencing. With the head-to-tail splice site as the target, we observed a consistent sequence between the Sanger sequencing results and circBase (Fig. 1F). RNase R, a ubiquitous 3′ exoribonuclease, has no effect on circRNAs. RNase R was added to the total RNA samples to further verify the circRNA nature of circDOCK1. This assay showed that circDOCK1 is truly a circRNA, as it was resistant to RNase R digestion (Fig. 1G). Then, cDNA and genomic DNA (gDNA) with or without RNase R treatment were amplified by convergent primers or divergent primers to amplify linear or circular DOCK1. The results showed that circDOCK1, which was observed in only cDNA amplified by divergent primers but not in gDNA, could resist RNase R treatment (Fig. 1H). The linear DOCK1 amplified by convergent primers was digested by RNase R (Fig. 1G-H). In addition, after treatment with the transcription inhibitor actinomycin D, the linear transcript of DOCK1 in MG63 and U2OS cell lines exhibited a shorter half-life than circDOCK1 (Fig. 1I). Nuclear and cytoplasmic RNAs were analyzed by qRT–PCR, which indicated that circDOCK1 was mainly localized in the cytoplasm (Fig. 1J), as demonstrated by FISH for circDOCK1 (Fig. 1K). In brief, these findings indicate that circDOCK1 is mainly localized in the cytoplasm, has good stability, and may be a participating factor in the occurrence and development of OS.

### CircDOCK1 promotes the malignant transformation of OS cells

**Fig. 2:**
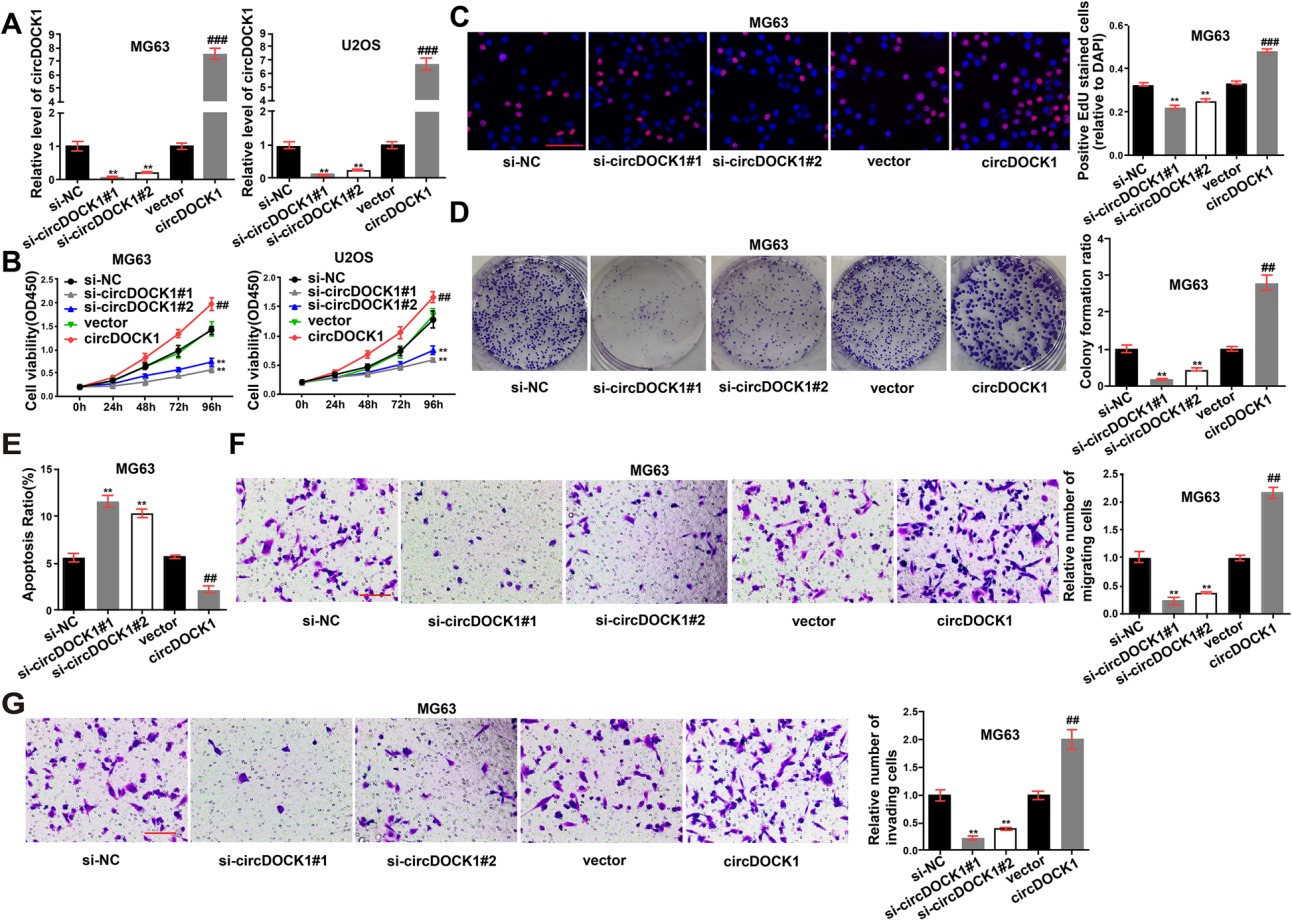
CircDOCK1 promotes the malignant transformation of OS cells. **A** circDOCK1 RNA expression was measured by qRT–PCR post transfection of the overexpression plasmid, siRNA1 and siRNA2 (normalized to si-NC or vector). **B** CCK-8 assay assessing MG63 and U2OS cell proliferation post transfection with circDOCK1 siRNAs or the overexpression plasmid. **C** EdU incorporation assays assessing MG63 cell proliferation (scale bar = 100 μm). **D** Colony formation assays (normalized to si-NC or vector). **E** Flow cytometry detecting the influence of circDOCK1 on cell apoptosis. **F-G** Transwell assays detecting the influence of circDOCK1 on cell migration (**F**) and invasion (**G**) (normalized to si-NC or vector, scale bar = 100 μm). The data are presented as the mean ± SD of three independent experiments. **P < 0.01, ***P < 0.001, *vs. si-NC, ^#^vs. vector

To further explore the influence of circDOCK1 on OS cells, the junction sites were targeted by two designed siRNAs (Table S1 in Additional file 1). RNA interference and overexpression plasmids were selected for subsequent experiments (Fig. 2A). The enhancement of OS cell proliferation by circDOCK1 overexpression was verified by EdU and CCK-8 assays (Fig. 2B-C, Fig. S2A in Additional file 2). Overexpression of circDOCK1 also increased colony formation (Fig. 2D, Fig. S2B in Additional file 2). Apoptosis was inhibited in circDOCK1-overexpressing cells and enhanced in cells expressing lower levels of circDOCK1 (Fig. 2E, Fig. S2C in Additional file 2). Additionally, decreased cell migration and invasion were observed in cells transfected with circDOCK1-specific siRNA (Fig. 2F-G, Fig. S2D-E in Additional file 2).

### CircDOCK1 serves as a miR-339-3p sponge

**Fig. 3:**
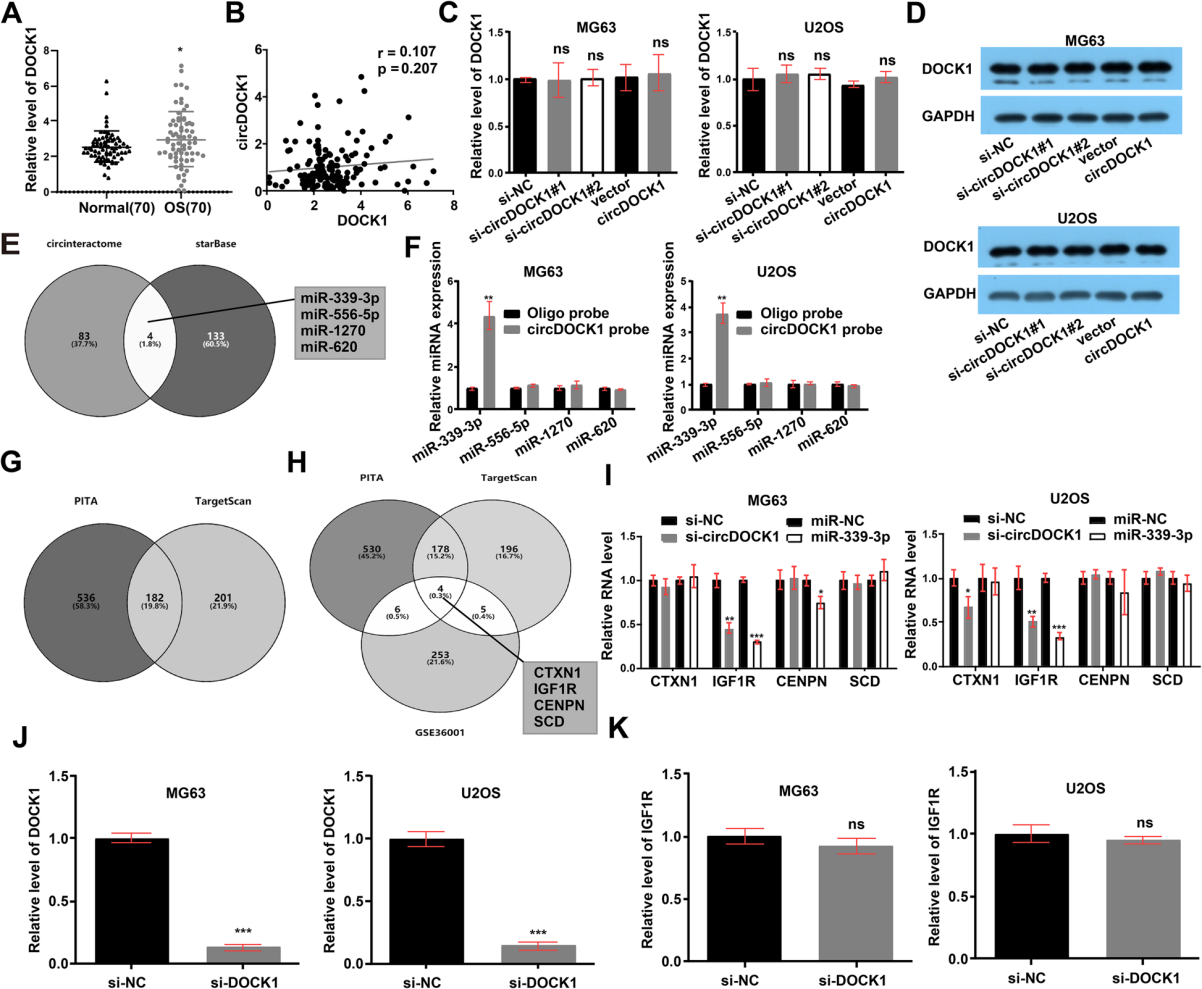
CircDOCK1 serves as a miR-339-3p sponge. **A** Linear DOCK1 expression in OS tissues. **B** Association between circDOCK1 and DOCK1 (*P* = 0.207). **C** Suppression and overexpression of circDOCK1 had no modulatory effect on linear DOCK1 RNA expression in OS cells (normalized to si-NC or vector). **D** Suppression and overexpression of circDOCK1 had no modulatory effect on linear DOCK1 protein expression in OS cells. **E** Prediction of potential target miRNAs possibly binding to circDOCK1 with CircInteractome and starBase. **F** After pulldown with a circDOCK1-specific probe, miRNA expression was measured in lysates of MG63 and U2OS cells (normalized to the oligo probe). **G** PITA and TargetScan predictions of the target genes of miR-339-3p. **H** Combination of the prediction results with the GSE36001 RNA microarray analysis results. **I** Expression of potential targets of miR-339-3p in MG63 and U2OS cells post transfection with the miR-339-3p mimic or circDOCK1 siRNA (normalized to si-NC or miR-NC). **J** DOCK1 RNA expression was measured by qRT–PCR post transfection of the siRNA (normalized to si-NC). **K** RNA expression of IGF1R in MG63 and U2OS cells post transfection with the DOCK1 siRNA (normalized to si-NC). The data are presented as the mean ± SD of three independent experiments. *P < 0.05, **P < 0.01, ***P < 0.001, ns: nonsignificant

However, the mechanism by which circDOCK1 is involved in OS progression is not yet known. CircDOCK1 is localized in the cytoplasm, suggesting that it may be implicated in the development of OS at the posttranscriptional level. To determine whether circDOCK1 can regulate parental gene expression, linear DOCK1 expression was measured in OS tissues. The qRT–PCR results showed higher DOCK1 expresison in OS tissues than in control tissues and no association with circDOCK1 expression (Fig. 3A-B). The mRNA and protein expression levels of linear DOCK1 did not change with suppression or overexpression of circDOCK1 (Fig. 3C-D). Then, we sought to determine whether circDOCK1 can interact with RBPs. Through bioinformatics analysis with CircInteractome, we predicted 9 RBPs that might interact with circDOCK1. Then, we verified the binding relationships with a RIP assay. The results showed that circDOCK1 was enriched in the AGO2 precipitate compared with the IgG precipitate (Fig. S3A in Additional file 2). Since miRNA-mediated gene silencing cannot be separated from AGO2-mediated gene silencing [21], we sought to determine whether circDOCK1 can function as a miRNA sponge.

StarBase and CircInteractome were used as tools to predict the candidate target miRNAs possibly binding to the circDOCK1 sequence (Fig. 3E). For circRNA pulldown experiments, a dedicated biotin-labeled circDOCK1 probe was used, and circDOCK1-related RNAs were purified prior to qRT–PCR. Based on the experimental results, compared with control group samples, samples pulled down with the circDOCK1-specific probe showed notable enrichment of only miR-339-3p (Fig. 3F). Next, TargetScan and PITA were used as tools to predict the target genes of miR-339-3p (Fig. 3G). The predictions were combined with the results of the RNA microarray analysis results of GSE36001 (Fig. S3B-C in Additional file 2), and four mRNAs were selected for further studies (Fig. 3H). Among these four potential target mRNAs, only IGF1R was regulated by circDOCK1 and miR-339-3p (Fig. 3I). To rule out DOCK1 interference, we included a DOCK1 knockdown condition in the current Fig. 3J. The result showed that IGFR1 level was indifferent to depletion of linear DOCK1 (Fig. 3K). CircDOCK1 may regulate IGF1R as a miR-339-3p sponge, as indicated by the above findings.

### The roles of circDOCK1 and IGF1R in vivo

**Fig. 4:**
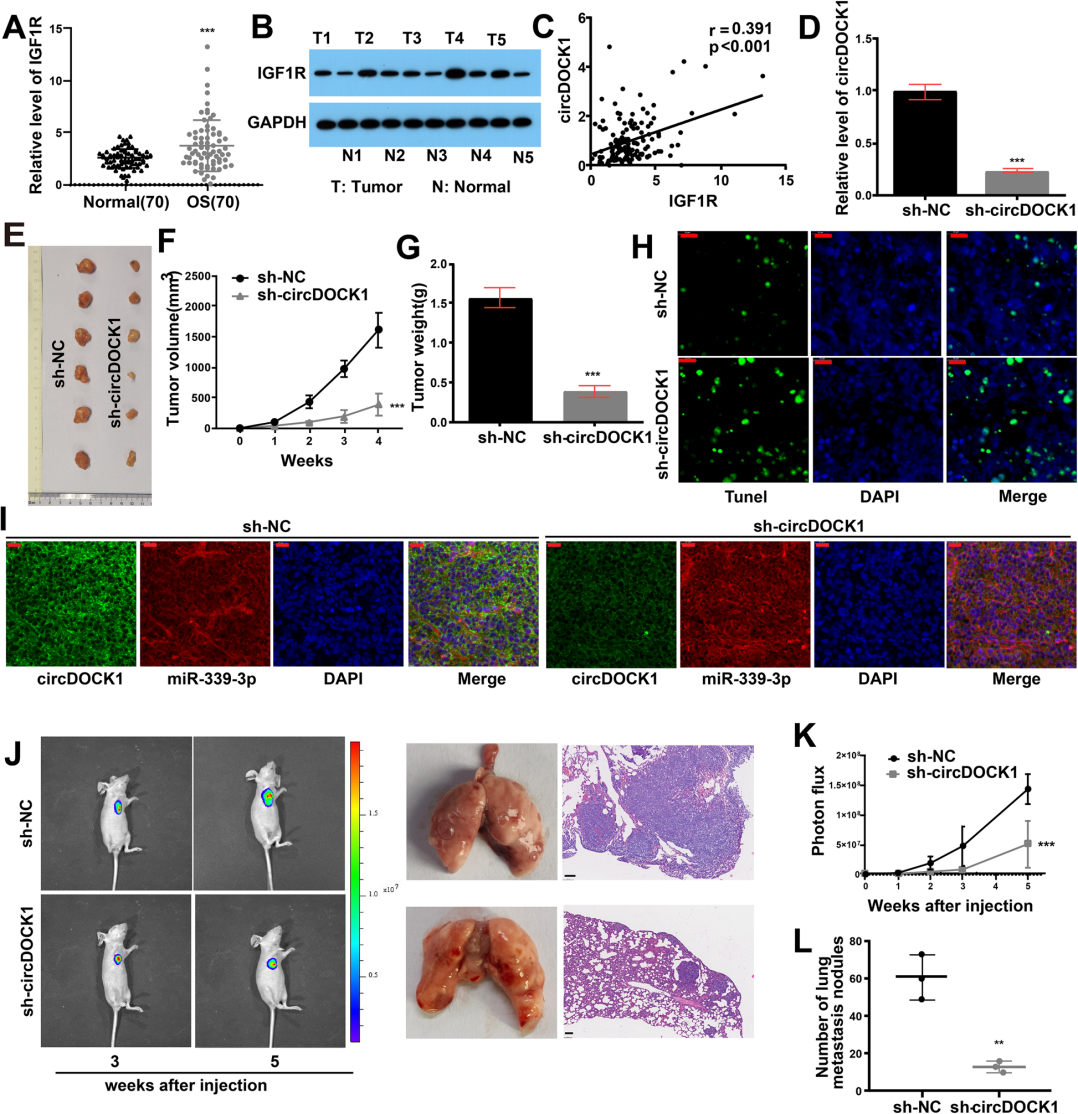
Roles of circDOCK1 and IGF1R in vivo. **A** qRT–PCR detecting the mRNA level of IGF1R in OS tissues. **B** Western blot analysis measuring the protein level of IGF1R in OS tissues. **C** Positive association between circDOCK1 expression and IGF1R expression (*P* < 0.001). **D** circDOCK1 RNA expression was measured by qRT–PCR post transfection of the shRNA (normalized to sh-NC). **E** Subcutaneous injection of MG63 cells into nude mice. **F** Tumor volumes. **G** Tumor weights. **H** TUNEL assay (scale bar = 20 μm). **I** FISH of miR-339-3p and circDOCK1 in xenograft tumors (scale bar = 20 μm). **J-K** Luciferase signal intensities and H&E staining post tail vein injection in nude mice (scale bar = 100 μm). **L** Number of metastatic nodules in the lungs post tail vein injection. The data are presented as the mean ± SD values. **P < 0.01, ***P < 0.001

To investigate the roles of circDOCK1 and IGF1R in vivo, we detected the expression of IGF1R in OS tissues and found that OS tissues had markedly higher mRNA and protein levels of IGF1R (Fig. 4A-B). Moreover, circDOCK1 expression was significantly positively correlated with IGF1R expression (Fig. 4C).

Furthermore, nude mice were inoculated subcutaneously with MG63 cells stably transfected with circDOCK1-shRNA or sh-NC and were closely monitored for 4 weeks for tumor growth. The results revealed that the circDOCK1-shRNA group had considerably lower tumor weights and volumes than the sh-NC group (Fig. 4D-G). Subsequently, we performed TdT-mediated dUTP Nick-End Labeling (TUNEL), FISH, qRT–PCR and IHC assays using xenograft tumors derived from nude mice. The findings revealed that the apoptosis levels in tumors from the circDOCK1-shRNA group were significantly higher than those in tumors from the sh-NC group (Fig. 4H). The FISH assay showed that both circDOCK1 and miR-339-3p were enriched in the cytoplasm (Fig. 4I). The decreased expression of IGF1R associated with circDOCK1 shRNA transfection was verified by both IHC and qRT–PCR (Fig. S4A-C in Additional file 2). In addition, the expression of Ki-67 was downregulated by circDOCK1 shRNA transfection (Fig. S4C in Additional file 2). Subsequently, MG63 cells were injected into nude mice via the tail vein to establish the lung metastasis model. On photon flux curves, notably fewer lung metastases were observed in the circDOCK1 shRNA group (Fig. 4J-K). Six weeks later, lung metastasis was found to be suppressed by downregulation of circDOCK1, as confirmed by hematoxylin and eosin (H&E) staining of excised lungs (Fig. 4J-L, Fig. S4D in Additional file 2).

### CircDOCK1 regulates malignant transformation via the miR-339-3p/IGF1R axis in vitro

**Fig. 5:**
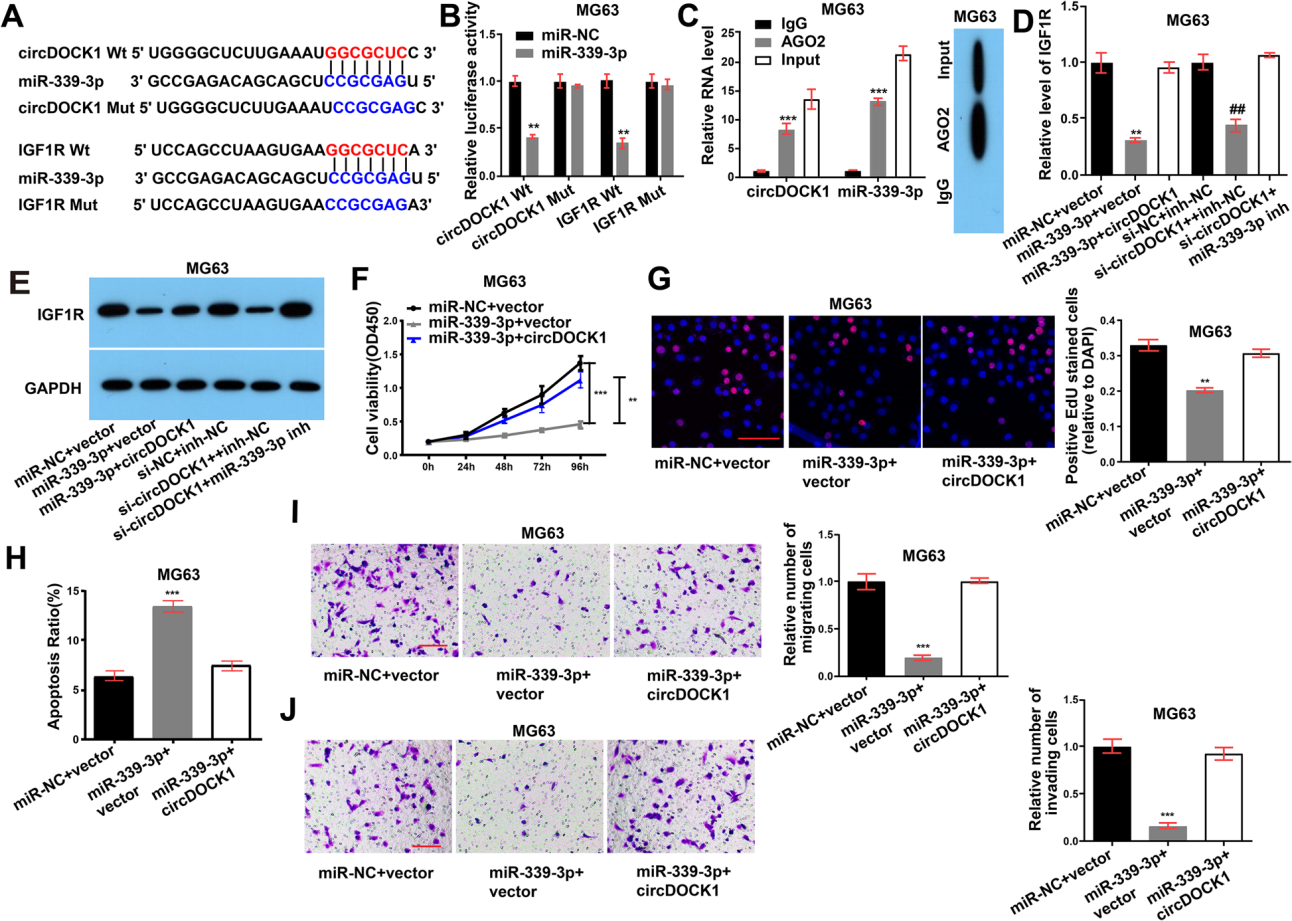
CircDOCK1 regulates malignant transformation via the miR-339-3p/IGF1R axis in vitro. **A** Hypothetical and mutant binding sites in miR-339-3p for IGF1R (lower) and circDOCK1 (upper). **B** Luciferase reporter assay detecting the binding of miR-339-3p to circDOCK1 and IGF1R in MG63 cell lines (normalized to miR-NC). **C** RIP with an anti-AGO2 antibody in the MG63 cell line evaluating the transcript levels of circDOCK1 and miR-339-3p (left). Western blot analysis evaluating the AGO2 protein level (right) (normalized to a control). **D-E** The mRNA (**D**) and protein (**E**) levels of IGF1R in MG63 cell lines post transfection (normalized to IgG). **F** CCK-8 assay. **G** EdU incorporation assay (scale bar = 100 μm). **H** Flow cytometric analysis of apoptosis. **I** Transwell migration assay (normalized to miR-NC + vector, scale bar = 100 μm). **J** Transwell invasion assay (normalized to miR-NC + vector, scale bar = 100 μm). The data are presented as the mean ± SD of three independent experiments. **P < 0.01, ***P < 0.001

Further in vitro experiments were performed to determine whether tumorigenesis and malignant transformation are regulated by the circDOCK1/miR-339-3p/IGF1R axis. Bioinformatics database analysis predicted that circDOCK1 and IGF1R bind to miR-339-3p, as expected (Fig. 5A). Additionally, the results of the luciferase reporter assay further indicated that miR-339-3p directly binds to a site in circDOCK1 and a site in the 3′-UTR of IGF1R (Fig. 5B, Fig. S5A in Additional file 2). RIP was then performed using an anti-AGO2 antibody in the MG63 and U2OS cell lines and showed that the amounts of circDOCK1 and miR-339-3p were higher in the anti-AGO2 precipitate than in the IgG precipitate (Fig. 5C, Fig. S5B in Additional file 2). Collectively, these findings suggest that circDOCK1 may sponge miR-339-3p.

Furthermore, we investigated the potential mechanism by which the circDOCK1/miR-339-3p axis regulates OS progression. The decreases in the mRNA and protein levels of IGF1R caused by miR-339-3p mimic or circDOCK1 siRNA transfection were reversed by overexpression of circDOCK1 or transfection with a miR-339-3p inhibitor, respectively (Fig. 5D-E, Fig. S5C-D in Additional file 2). In addition, overexpression of miR-339-3p in MG63 and U2OS cells reduced proliferation (Fig. 5F-G, Fig. S5E-F in Additional file 2), migration, and invasion (Fig. 5I-J, Fig. S5H-I in Additional file 2) but increased apoptosis (Fig. 5H, Fig. S5G in Additional file 2). To further identify whether circDOCK1 exerts its effects by interacting with miR-339-3p, the miR-339-3p mimic and circDOCK1 expression plasmid were cotransfected into OS cells. Cotransfection with the circDOCK1 expression plasmid showed the opposite effects compared with the miR-339-3p-induced effects on the growth, apoptosis and motility of OS cells (Fig. 5F-J, Fig. S5E-I in Additional file 2). These findings suggest that circDOCK1 regulates malignant transformation by sponging miR-339-3p.

### CircDOCK1 regulates the sensitivity of OS cells to cisplatin

**Fig. 6:**
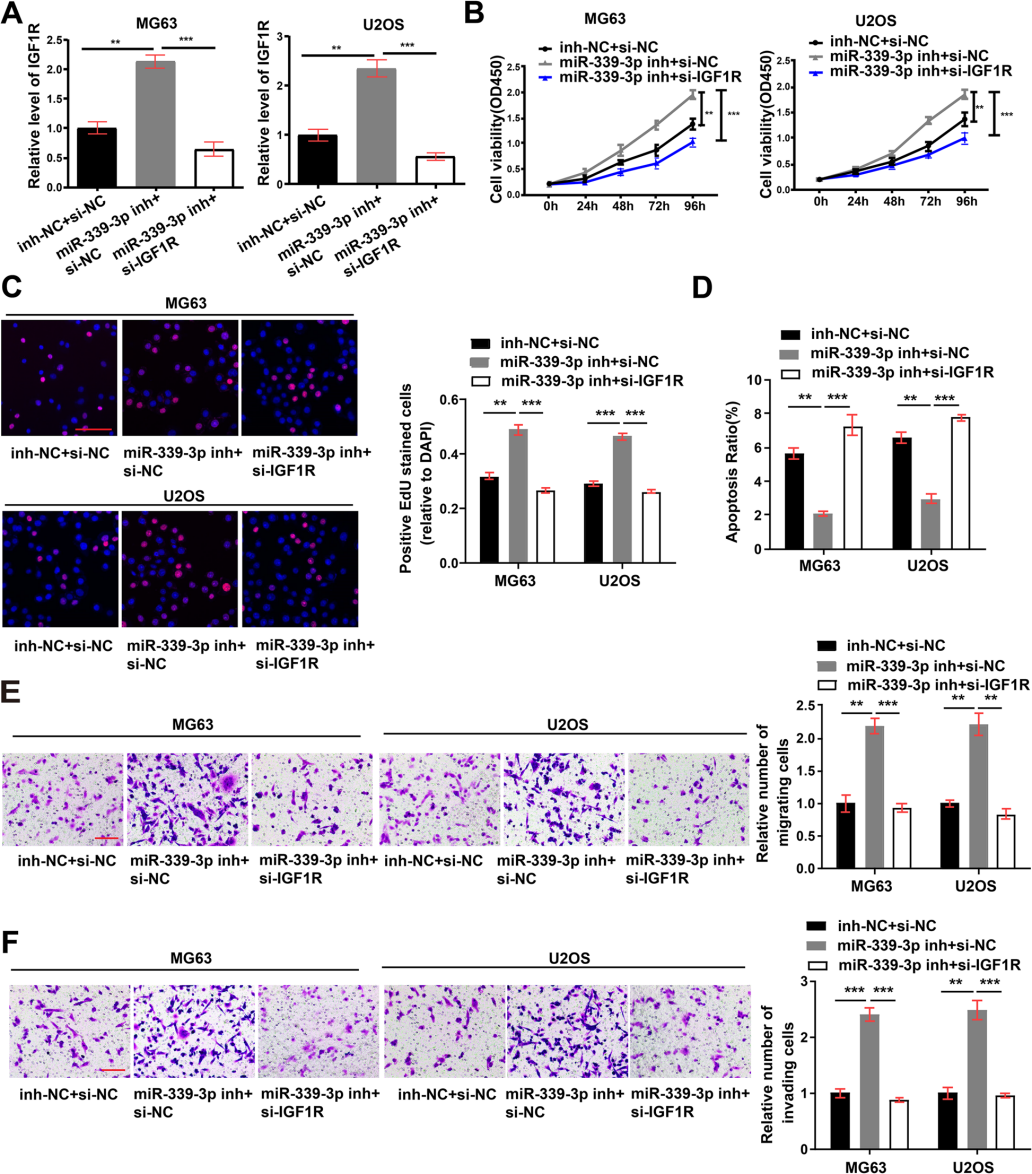
Roles of IGF1R in vitro. **A** mRNA expression of IGF1R in cells post cotransfection with IGF1R siRNA and the miR-339-3p inhibitor (normalized to inh-NC + si-NC). **B-F** CCK-8 (**B**), EdU incorporation (scale bar = 100 μm) (**C**), flow cytometry (**D**), Transwell migration (normalized to inh-NC + si-NC, scale bar = 100 μm) (**E**) and Transwell invasion (normalized to inh-NC + si-NC, scale bar = 100 μm) (**F**) assays measuring the migration, proliferation, apoptosis and invasion of cells post cotransfection with IGF1R siRNA and the miR-339-3p inhibitor. The data are presented as the mean ± SD of three independent experiments. **P < 0.01, ***P < 0.001

To fully confirm the circDOCK1/miR-339-3p/IGF1R axis, we cotransfected OS cells with the miR-339-3p inhibitor and IGF1R siRNA. The level of IGF1R was increased in the miR-339-3p inhibitor group, while cotransfection with IGF1R siRNA restored the IGF1R level (Fig. 6A). EdU, CCK-8, Transwell migration and invasion assays and flow cytometric analysis revealed that decreasing the expression of IGF1R restored the effects on cell proliferation, migration, apoptosis and invasion induced by the miR-339-3p inhibitor (Fig. 6B-F).

**Fig. 7:**
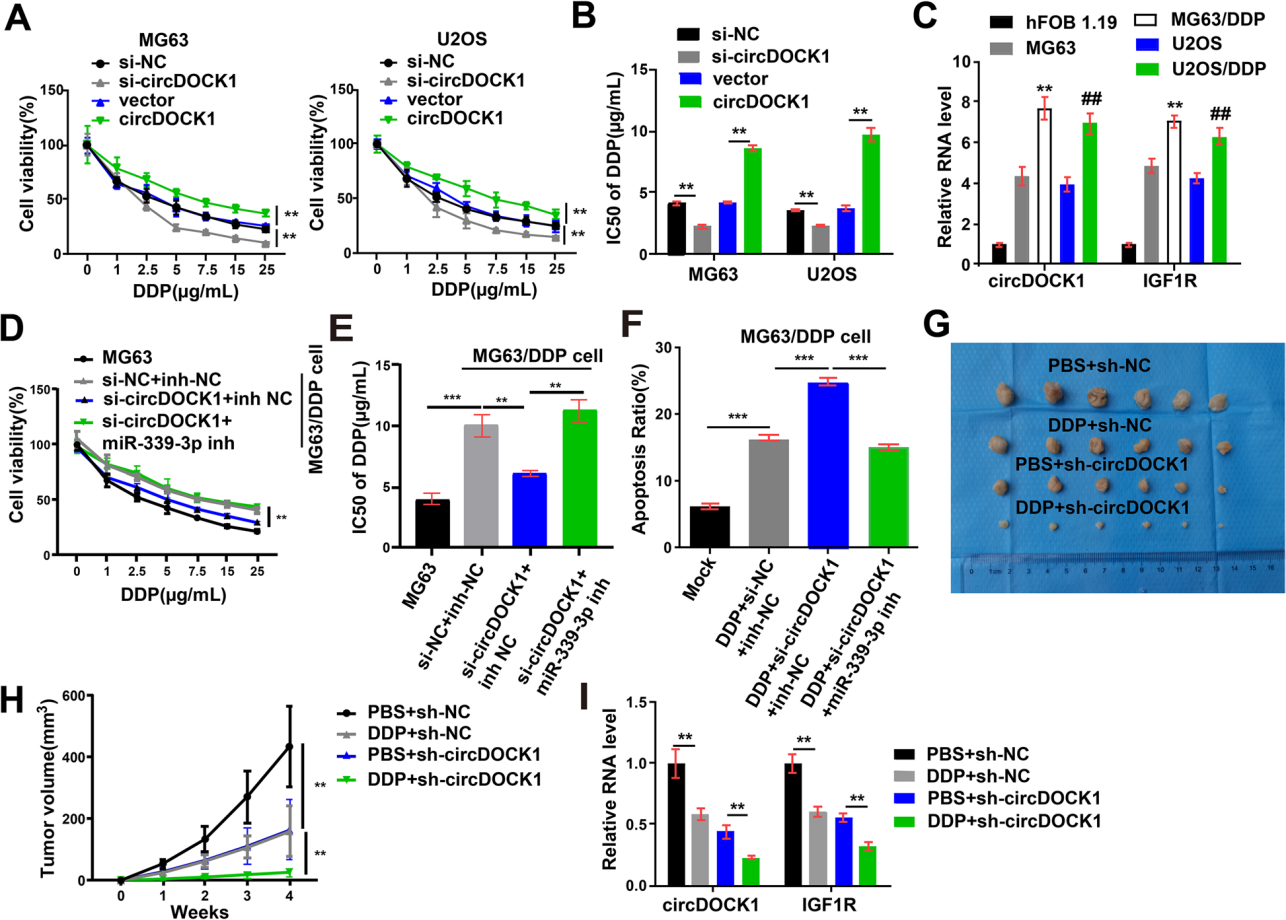
CircDOCK1 regulates the sensitivity of OS cells to cisplatin. **A** Relative viability of OS cells after 48 h of treatment with cisplatin at the specified concentrations. **B** The IC50 of OS cells treated with cisplatin. **C** The transcript levels of circDOCK1 and IGF1R in the hFOB 1.19, MG63, MG63/DDP, U2OS and U2OS/DDP cell lines (normalized to hFOB 1.19 cells). **D** Cell growth of MG63 cells and MG63/DDP cells after 48 h of treatment with cisplatin at the specified concentrations. **E** The IC50 of MG63 cells and MG63/DDP cells treated with cisplatin. **F** Cell apoptosis of MG63/DDP cells cotransfected with the miR-339-3p inhibitor and circDOCK1 siRNA. **G** Xenograft tumors from euthanized mice with or without cisplatin treatment. **H** Volumes of xenograft tumors. **I** The transcript levels of IGF1R and circDOCK1 in xenograft tumors (normalized to PBS + sh-NC). The data are presented as the mean ± SD of three independent experiments. **P < 0.01, ***P < 0.001

A series of studies have indicated that IGF1R is closely related to tumor drug resistance [22–24]. We sought to determine whether circDOCK1 is involved in the chemoresistance of OS cells and performed experiments with cisplatin and DOX. The results revealed that decreased circDOCK1 expression markedly reduced the viability of cisplatin/DOX-treated cells, whereas increased circDOCK1 expression markedly induced cisplatin/DOX resistance (Fig. 7A, Fig. S6A in Additional file 2). The IC50 was also decreased by circDOCK1-specific siRNA and increased by transfection of the circDOCK1 overexpression plasmid (Fig. 7B).

To test the hypothesis that circDOCK1 can regulate sensitivity to cisplatin, cisplatin-resistant MG63/DDP and U2OS/DDP cell lines were generated. In comparison with the paired normal OS cell lines, the cisplatin-resistant OS cell lines exhibited markedly increased circDOCK1 and IGF1R expression (Fig. 7C). After cotransfection with the miR-339-3p inhibitor and circDOCK1 siRNAs, cisplatin was used for the treatment of the MG63/DDP and U2OS/DDP cell lines. The effects of the circDOCK1 siRNA on proliferation and apoptosis were reversed by cotransfection with the miR-339-3p inhibitor (Fig. 7D-F, Fig. S7A-B in Additional file 2).

For the in vivo experiment, nude mice were injected with U2OS cells stably transfected with circDOCK1-shRNA or sh-NC, and tumors were allowed to grow for 4 weeks. The data suggested that downregulation of circDOCK1 considerably reduced the growth of xenograft tumors and made cells sensitive to cisplatin (Fig. 7G-H, Fig. S7C in Additional file 2). Moreover, downregulation of circDOCK1 markedly reduced the transcript levels of circDOCK1 and IGF1R, as determined by qRT–PCR (Fig. 7I), and notably reduced the protein level of IGF1R, as determined by IHC staining of xenograft samples (Fig. S7D in Additional file 2). The above findings reveal that downregulation of circDOCK1 promotes cisplatin sensitivity by sponging miR-339-3p.

## Discussion

Over the last 30 years, the 5-year survival rate of OS patients has increased; however, metastatic or drug-resistant OS remains a challenge [25, 26]. Clinically, effective new treatment targets for refractory OS are required.

In recent years, with the development of bioinformatics and high-throughput sequencing technologies, increasing attention has been given to circRNAs [9, 27–30]. As a result of their distinct characteristics, including their unique structure, cell type-specific and tissue-specific expression, conservation across species, and stable expression in exosomes, blood and saliva [31–35], more than 10,000 different circRNAs have been discovered and researched in various organisms, which has also increased research focus on circRNAs in malignancy [28, 35–37]. At present, the differential expression of circRNAs in OS tissues has been identified in many studies [2, 19, 38]. Our bioinformatics analysis and experimental verification showed high expression of circDOCK1 in OS tissues and cell lines, suggesting that circDOCK1 may be a regulator of OS progression. Moreover, circRNAs are involved in all pathophysiological processes involved in OS development and treatment, including proliferation, apoptosis and chemoresistance [39–41]. Our study revealed that overexpression of circDOCK1 promoted the proliferation, migration, invasion and chemoresistance of OS cells in vitro and inhibited apoptosis, as validated in xenograft and lung metastasis experiments in vivo. In addition, in bladder carcinoma, circDOCK1 was identified to promote tumor progression by modulating the circDOCK1/hsa-miR-132-3p/Sox5 signaling axis [42]. Similarly, in oral squamous cell carcinoma, circDOCK1 was identified to suppress cell apoptosis via inhibition of miR-196a-5p by targeting BIRC3 [43].

Accumulating evidence indicates that circRNAs exert their effects mainly through three mechanisms: (1) cis regulation of parental gene expression; (2) miRNA sponging to regulate gene expression (i.e., acting as competitive endogenous RNAs); and (3) formation of complexes with RBPs [10]. Here, we first excluded the effect of circDOCK1 on its parental gene. Then, we predicted 9 RBPs that might interact with circDOCK1. By a RIP assay, we found that only AGO2 can bind to circDOCK1. As AGO2 is very important for miRNA-mediated gene silencing [21], we suspected that circDOCK1 may function as a miRNA sponge. In this study, miR-339-3p was identified as the potential target miRNA of circDOCK1 by bioinformatics analysis. Then, the binding relationship was verified by a circRNA pulldown assay. In melanoma, miR-339-3p is a tumor inhibitor [44]. In colorectal cancer, miR-339-3p is reported to suppress proliferation and metastasis [45]. However, the role of miR-339-3p in OS is still unclear. Here, luciferase reporter, RIP and FISH assays were conducted to verify the direct interaction of circDOCK1 and miR-339-3p. Furthermore, it was predicted that IGF1R was one of the miR-339-3p target genes. Then, the gene expression pattern in 19 OS cell lines and 6 normal samples (GSE36001) was analyzed to identify target genes. Finally, the association between IGF1R and miR-339-3p was confirmed by dual-luciferase reporter and RIP assays. IGF1R is a heterotetrameric transmembrane glycoprotein. After binding to a ligand, IGF1R is autophosphorylated, which activates its tyrosine kinase function, and it then interacts with adaptor molecules such as insulin receptor substrates and Shc, thus activating downstream protein kinases, including those in the PI3K/AKT and MAPK/ERK1/2 signaling pathways that regulate the growth and survival of cancer cells [46]. IGF1R is reported to be closely related to chemoresistance [22–24]. In addition, IGF1R has been identified as an oncogene in the pathogenesis of OS [47–49]. IGF1R is also reported to serve as a potential target for the treatment of high-grade OS [50]. Moreover, IGF1R suppression enhances the response to doxorubicin chemotherapy in some OS cell lines [51]. In this study, high expression of IGF1R was identified in OS tissues, and its role in OS and the correlation with circDOCK1 were verified in vitro and in vivo.

Cisplatin is one of the major chemotherapeutic agents for OS patients [52, 53]; however, chemoresistance is the main cause of the unfavorable prognosis of OS patients [54, 55]. Recently, studies have shown that circRNAs are crucial in regulating the cisplatin sensitivity of OS cells. CircPVT1 is involved in the resistance of OS cells to doxorubicin and cisplatin through the regulation of ABCB1 [56]. CircUBAP2 promotes SEMA6D expression to increase the cisplatin resistance of OS cells [57]. This study showed that circDOCK1 has regulatory effects on cisplatin sensitivity in vivo and in vitro. In numerous cancers, including OS, IGF1R may promote the chemotherapeutic resistance of tumor cells [22, 24, 58]. This study signified that circDOCK1 may affect the cisplatin sensitivity of OS cells by regulating the miR-339-3p/IGF1R axis.

## Conclusions

In summary, circDOCK1 overexpression was observed in OS tissues and cell lines and promoted OS tumorigenesis, probably by sponging miR-339-3p to regulate IGF1R in vivo and in vitro. Furthermore, circDOCK1 regulated cisplatin sensitivity via the miR-339-3p/IGF1R axis. Collectively, our results indicate that the circDOCK1/miR-339-3p/IGF1R axis may be a therapeutic target and the key mechanism in OS.

## Supplementary Information

**Additional file 1: Table S1.** Sequences of primers used to construct the transfectants. **Table S2.** Sequences of primers used for qRT–PCR. **Table S3.** Associations between circDOCK1 expression and the clinicopathological characteristics of OS patients.

**Additional file 2: Figure S1.** A: Volcano plot of genes in the GSE140256 dataset. B: Heatmap of the circRNA microarray (GSE140256) analysis results showing 10 dysregulated circRNAs, including 4 upregulated circRNAs, in OS tissues (log2FC > 1 or < − 1, *P* value < 0.05). C: CircRNA expression in OS cells. **Figure S2.** A: EdU incorporation assays and B: colony formation assays assessing the proliferation of U2OS cells transfected with circDOCK1-specific siRNAs or overexpression plasmids. C: Flow cytometric analysis assessing U2OS cell apoptosis. D-E: Transwell assay assessing the migration (D) and invasion (E) of U2OS cells. **Figure S3.** A: RIP assays detecting the binding between circDOCK1 and the 9 predicted RBPs. B: Heatmap of the RNA microarray (GSE36001) analysis results. C: Volcano plot of genes in the GSE36001 dataset. **Figure S4.** A-B: The transcript levels of circDOCK1 (A) and IGF1R (B) in xenograft tumors. C: The IGF1R and Ki-67 protein levels in xenograft tumors, as evaluated by IHC. D: H&E staining of mouse lungs after tail vein injection. **Figure S5.** A: Luciferase reporter assay detecting the binding of miR-339-3p to circDOCK1 and IGF1R in U2OS cell lines. B: CircDOCK1 and miR-339-3p transcript levels (left) as determined by RIP with an anti-AGO2 antibody in the U2OS cell line and AGO2 protein levels as determined by western blotting (right). C-D: Cotransfection of the miR-339-3p mimic and circDOCK1 overexpression plasmid or miR-339-3p inhibitor and circDOCK1 siRNA to measure the mRNA (C) and protein (D) levels of IGF1R in U2OS cell lines. E-I: Cotransfection of the miR-339-3p mimic and circDOCK1 overexpression plasmid to evaluate malignant transformation by CCK-8 (E), EdU incorporation (F), flow cytometry (G), Transwell migration (H) and Transwell invasion (I) assays in the U2OS cell line. **Figure S6.** A: Relative viability of circDOCK1 overexpression plasmid- or siRNA-transfected cells after 48 h of exposure to DOX at the specified concentrations. **Figure S7.** A-B: Cell growth (A) and apoptosis (B) in U2OS/DDP cell lines cotransfected with the miR-339-3p inhibitor and circDOCK1 siRNA. C: Tumor weights. D: IGF1R protein levels in xenograft tumors, as evaluated by IHC.

## Data Availability

The datasets used and/or analyzed during the current study are available from the corresponding author on reasonable request.

## Abbreviations

OS: Osteogenic sarcoma
ncRNAs: Noncoding RNAs
circRNA: Circular RNA
miRNA: MicroRNA
IGF1R: Insulin-like growth factor 1 receptor
FISH: Fluorescence in situ hybridization
AUC: Area under the ROC curve
CCK-8: Cell Counting Kit-8
RIP: RNA immunoprecipitation
H&E: Hematoxylin and eosin
FBS: Fetal bovine serum
EdU: 5-Ethynyl-2′-deoxyuridine
DAPI: 4′,6-Diamidino-2-phenylindole
RIPA: Radioimmunoprecipitation assay
RBPs: RNA binding proteins
RNase R: Ribonuclease R
DDP: Cisplatin
DOX: Doxorubicin hydrochloride
qRT–PCR: Quantitative real-time polymerase chain reaction
TUNEL: TdT-mediated dUTP Nick-End Labeling
