# Supplementary material for: CircDOCK1 promotes the tumorigenesis and cisplatin resistance of osteogenic sarcoma via the miR-339-3p/IGF1R axis
Source: Mol Cancer. 2021 Dec 7;20:161. doi: 10.1186/s12943-021-01453-0 (PMC8650521; doi:10.1186/s12943-021-01453-0)
Supplement: Supplementary file 1 — Additional file 1: Table S1. Sequences of primers used to construct the transfectants. Table S2. Sequences of primers used for qRT–PCR. Table S3. Associations between circDOCK1 expression and the clinicopathological characteristics of OS patients. [file 12943_2021_1453_MOESM1_ESM.docx]

**Table S1.** Sequences of primers used to construct the transfectants.

| **Name** |  | **Sequence** |
| --- | --- | --- |
| circDOCK1 shRNA | Sense  Anti-sense | 5’-CCGGATTCTGAACTCATTGAATGGA  CTCGAGTCCATTCAATGAGTTCAGAATTTTTTG-3’  5’-AATTCAAAAAATTCTGAACTCATTGAATGGA  CTCGAGTCCATTCAATGAGTTCAGAAT-3’ |
| shRNA NC | Sense | 5’-CCGGCAACAAGATGAAGAGCAC  CAACTCGAGTTGGTGCTCTTCATCTTGTTGTTTTTG-3’ |
|  | Anti-sense | 5’-AATTCAAAAACAACAAGATGA  AGAGCACCAACTCGAGTTGGTGCTCTTCATCTTGTTG-3’ |
| circDOCK1 siRNA1  circDOCK1 siRNA2 | Sense  Anti-sense  Sense  Anti-sense | 5’-UCUGAACUCAUUGAAUGGATT-3’  5’-UCCAUUCAAUGAGUUCAGATT-3’  5’-CUGAACUCAUUGAAUGGAATT-3’  5’-UUCCAUUCAAUGAGUUCAGTT-3’ |
| siRNA NC | Sense | 5’-UUCUCCGAACGUGUCACGUTT-3’ |
|  | Anti-sense | 5’-ACGUGACACGUUCGGAGAATT-3’ |
| miR-339-3p mimics | Sense | 5’-UGAGCGCCUCGACGACAGAGCCG-3’ |
| mimics NC | Anti-sense  Sense | 5’-GCUCUGUCGUCGAGGCGCUCAUU-3’  5’-UUCUCCGAACGUGUCACGUTT-3’ |
|  | Anti-sense | 5’-ACGUGACACGUUCGGAGAATT-3’ |
| miR-339-3p inhibitor |  | 5’-CGGCUCUGUCGUCGAGGCGCUCA-3’ |
| inhibitor NC |  | 5’-CAGUACUUUUGUGUAGUACAA-3’ |
| DOCK1 siRNA | Sense | 5’-GAUGAACUUUCUUUACAGATT-3’ |
|  | Anti-sense | 5’-UCUGUAAAGAAAGUUCAUCTT-3’ |
| IGF1R siRNA | Sense | 5’-GCAAUUUGCUCAUUAACAUTT-3’ |
|  | Anti-sense | 5’-AUGUUAAUGAGCAAAUUGCTT-3’ |

| **Name** | **Sequence** |
| --- | --- |
| circDOCK1 | F: 5’- CCAGAGGCACGTCCAGATTA -3’  R: 5’- AGGAAACTCCGCGTCTAGG -3’ |
| DOCK1 | F: 5’- ACCGAGGTTACACGTTACGAA -3’ |
|  | R: 5’- TCGGAGTGTCGTGGTGACTT -3’ |
| IGF1R | F:5’- TCGACATCCGCAACGACTATC -3’  R:5’- CCAGGGCGTAGTTGTAGAAGAG -3’ |
| U6 | F:5’- CTCGCTTCGGCAGCACA -3’ |
|  | R:5’- AACGCTTCACGAATTTGCGT -3’ |
| GAPDH | F:5’- CTGGGCTACACTGAGCACC -3’  R:5’- AAGTGGTCGTTGAGGGCAATG -3’ |

**Table S2.** Sequences of primers used for qRT–PCR.

**Table S3.** Associations between circDOCK1 expression and the clinicopathological characteristics of OS patients.

| **Feathers** | **Number** | **High** | **Low** | **P value** |
| --- | --- | --- | --- | --- |
| All cases | 70 | 28 | 42 |  |
| Age(years) |  |  |  | 0.806 |
| < 18 | 40 | 17 | 23 |  |
| ≥ 18 | 30 | 11 | 19 |  |
| Gender |  |  |  | 0.466 |
| Male | 33 | 15 | 18 |  |
| Female | 37 | 13 | 24 |  |
| Tumor size (cm) |  |  |  | **0.030** |
| < 5 | 34 | 9 | 25 |  |
| ≥ 5 | 36 | 19 | 17 |  |
| Distant metastasis |  |  |  | **0.028** |
| Absent | 37 | 10 | 27 |  |
| Present | 33 | 18 | 15 |  |
| Anatomic location |  |  |  | 0.632 |
| Tibia/femur | 40 | 15 | 25 |  |
| Elsewhere | 30 | 13 | 17 |  |
| Clinical stage |  |  |  | **0.008** |
| I~IIA | 39 | 10 | 29 |  |
| IIB~III | 31 | 18 | 13 |  |

Total data from 70 tumor tissues of osteogenic sarcoma patients were analyzed. For the expression of circDOCK1 was assayed by qRT-PCR, the average expression level was used as the cutoff. Data were analyzed by chi-squared test or Fisher’s exact test. P-value in bold indicates statistically significant.
